# P-1560. The 5XFAD Mouse Model of Alzheimer’s Disease Displays Delayed Recovery and Persistent Neuroinflammation Post-Clostridioides difficile Infection

**DOI:** 10.1093/ofid/ofaf695.1740

**Published:** 2026-01-11

**Authors:** Ashley K Nguyen, Suemin E Yang, Deiziane Costa, Maria L G Morais, Cirle A Warren

**Affiliations:** University of Virginia, Charlottesville, VA; University of Virginia, Charlottesville, VA; University of Virginia, Charlottesville, VA; University of Virginia, Charlottesville, VA; University of Virginia, Charlottesville, VA

## Abstract

**Background:**

*Clostridioides difficile* (*C. difficile*) is the primary cause of hospital- and community-acquired diarrhea associated with antibiotic use worldwide. *C. difficile* infection (CDI) affects nearly half a million patients in the USA annually, particularly the elderly. Previous studies have suggested that delirium and dementia may be associated with CDI and influence outcomes in humans. The mechanism of how dementia contributes to disease severity remains unknown. Here, we investigated whether Alzheimer’s disease (AD)-associated amyloid pathology (5xFAD mouse model) influences the clinical outcome of CDI, as well as if CDI impacts AD associated brain neuroinflammation.

**Methods:**

Wild-type (WT) and 5xFAD mice (5 months old) were infected with *C. difficile* (VPI 10463, oral gavage), monitored daily for weight loss, clinical symptoms, and *C. difficile* shedding for 75 days post-infection (pi). *C. difficile* shedding was determined by PCR. Inflammatory biomarker levels (MPO, Lcn2, IL-23, IL-33, IL-6, IL-1β, and TNF-α) in the cecum, prefrontal cortex, and hippocampus where then measured on day 7 and day 75 pi.

**Results:**

5xFAD infected mice had significantly worse diarrhea and higher total clinical score compared to WT infected mice on day 4 pi although no significant differences in *C. difficile* shedding in stools were detected. Additionally, the 5xFAD infected mice also appeared to recover slower from body weight loss. 5xFAD infected mice had elevated levels of IL-6, IL-23, and TNF-α in the cecum compared to WT infected mice on day 7 pi. Although 5xFAD infected and WT infected mice exhibited similar levels of inflammatory markers in the prefrontal cortex and hippocampus on day 7 pi, 5xFAD infected mice displayed a significant increase in MPO in the prefrontal cortex and increasing trend of Lcn2 synthesis in the hippocampus compared to WT infected mice on day 75 pi.

**Conclusion:**

Our data suggests that disease severity and gut inflammation during acute infection and neuroinflammation post-infection are increased in an amyloid pathology model of Alzheimer's disease. Further investigations are needed to better understand the interaction between CDI-induced inflammatory response and amyloid neuropathology.

**Disclosures:**

All Authors: No reported disclosures

